# A darwinian perspective: right premises, questionable conclusion. A commentary on Niall Shanks and Rebecca Pyles' "Evolution and medicine: the long reach of "Dr. Darwin""

**DOI:** 10.1186/1747-5341-3-6

**Published:** 2008-02-12

**Authors:** Paolo Vineis, Ronald Melnick

**Affiliations:** 1 Department of Epidemiology and Community Health, Imperial College London, St Mary's Campus, Norfolk Place, W2 1PG, London, UK; 2 Environmental Toxicology Program, National Institute of Environmental Health Sciences, Research Triangle Park, NC 27709, USA

## Abstract

As Dobzhansky wrote, nothing in biology makes sense outside the context of the evolutionary theory, and this truth has not been sufficiently explored yet by medicine. We comment on Shanks and Pyles' recently published paper, *Evolution and medicine: the long reach of "Dr. Darwin"*, and discuss some recent advancements in the application of evolutionary theory to carcinogenesis. However, we disagree with Shanks and Pyles about the usefulness of animal experiments in predicting human hazards. Based on the darwinian observation of inter-species and inter-individual variation in all biological functions, Shanks and Pyles suggest that animal experiments cannot be used to identify hazards to human health. We claim that while the activity of enzymes may vary among individuals and among species, this does not indicate that critical events in disease processes occurring after exposure to hazardous agents differ qualitatively between animal models and humans. In addition, the goal is to avoid human disease whenever possible and with the means that are available at a given point in time. Epidemics of cancer could have been prevented if experimental data had been used to reduce human exposures or ban carcinogenic chemicals. We discuss examples.

## 

The paper by Shanks and Pyles [1] correctly summarizes the contributions of evolutionary biology to medicine, following several other authors in different fields of medicine [2-5]. Carcinogenesis, for example, can be interpreted as the consequence of selection of mutated cells similar to that which, in the theory of evolution, occurs at the population level. Instead of considering a population of organisms, we can refer to a population of cells belonging to a multicellular organism. Cancer can be described as the outcome of mutation and selection. The rapid change in risk for some cancers after migration from one population to another suggests that carcinogenesis involves – in addition to mutations – some late event that most likely consists in the selection of cells already carrying mutations. To give a couple of examples, in the case of gastric and liver cancers, exposure to Helicobacter pylori (stomach cancer) or to hepatitis B or C viruses (liver cancer) in childhood would set the risk typical of the areas of origin, while reduced exposure to cofactors might help to explain the lower risk in those who migrate to Western countries. In the case of liver cancer in particular, a reduction in exposure to aflatoxins is believed to explain reduction in risk after migration. Cofactors can be interpreted as "selectogens", i.e. exposures that facilitate selection of mutated cells (cells previously exposed to "mutagens" or created by spontaneous replication errors).

This relatively new paradigm in carcinogenesis can thus be useful in understanding some observations made by epidemiologists, in particular the fact that many human carcinogenic exposures – such as hormones or some dietary constituents – are apparently not mutagenic.

Also, quite relevant to recent developments in the study of chronic diseases like cancer or cardiovascular disease is the idea that these are not fixed entities but rather constantly change their microscopic and clinical phenomenology, such that myocardial infarction today is a different entity from what it was 50 years ago, and lung cancer is now predominantly represented by adenocarcinomas instead of squamous cell carcinomas. This is in line with Darwin's fight against "fixism", or the "typological" view that interpreted species as fixed types.

Another consequence of evolutionary change and selection is that darwinism has induced the development of a number of powerful tools, in particular mathematical ones, to describe the competition among species and the concept of "fitness". "Darwinian dynamics" has become a very fruitful field of research, with applications in disparate disciplines from zoology to carcinogenesis [6]. Finally, but this is an incomplete list, therapeutics has at least in part taken advantage of darwinian concepts when dealing with resistance to antibiotics or to chemotherapy. It is perfectly justified to state, as Dobzhansky wrote, that nothing in biology makes sense outside the context of the evolutionary theory, and this truth has not been sufficiently explored yet by medicine.

Unfortunately, Shanks and Pyles derive from their darwinian approach some considerations relevant to disease prevention and public health that are not totally warranted. Public health is based on science but is more than science and involves policy decisions based on incomplete data, similarly to clinical medicine. In both, the end is not knowledge, but rather the well-being of the population (or the individual) and equity. Based on the darwinian observation of inter-species and inter-individual variation in all biological functions, Shanks and Pyles suggest that animal experiments are not very useful in identifying hazards to human health. They note large variations in response to drugs in both animals and humans, and call for the development of more individualized approaches to therapies. This is not particularly new and is certainly acceptable. However, they apply the same concept to the usefulness of animals in identifying potential human carcinogens, and here we disagree. The reasons for disagreement are essentially two-fold. First, prevention has an ethical component, i.e. the goal is to avoid human disease whenever possible and with the means that are available at a given point in time. Epidemics of cancer have occurred that could have been prevented if experimental data had been used to reduce human exposures or ban carcinogenic chemicals [7]. There are many reasons why we cannot rely upon observation in humans: it implies entails following-up cohorts of exposed subjects for decades, thus postponing prevention; often the populations are too small, or difficult to recruit and investigate, or exposure assessment is too complex. A very clear example is 1,3-butadiene, the carcinogenicity of which has been considered by a Working Group of the IARC Monographs [8]. More than 20 years ago one of us contributed to the first experiments showing that this widely used chemical induced cancers at multiple organ sites in rodents, including a very high incidence of otherwise extremely rare cancers (heart hemangiosarcomas). There was no doubt that the chemical was a potent carcinogen, given the consistency of the observations, the dose-response relationship, the unusual type of tumours, and the very high incidence. After so many years, however, still we have a limited number of sound epidemiological studies, given the considerable difficulties encountered in these kinds of investigations, but the studies are quite consistent with the animal observations.

Another argument against Shanks and Pyles' interpretation is the extremely successful use of animal experiments for preventive purposes, as clearly demonstrated by the history of the IARC Monographs.

Shanks and Pyles note that while humans and rodents used in biomedical research share numerous genetic similarities, allelic differences exist and rodent models used to evaluate diseases induced in humans typically have less genetic variability than do human populations. We certainly agree that the range of human variability due to polymorphisms at specific genetic loci cannot be captured from studies in animals of limited genetic variability. However, this limitation does not mean that rodents are not useful or relevant to the evaluation of diseases, such as cancer, in humans. For the most part, differences in how laboratory animals and humans metabolize environmental agents, or in the interactions of these agents with molecular targets (e.g., DNA, enzymes, or nuclear receptors), are quantitative in nature. For example, cytochrome P450 2E1, which is present in animals and humans, activates numerous environmental agents [9], including the known human carcinogens vinyl chloride and 1,3-butadiene, to DNA-reactive alkylating agents. While the activity of this enzyme and other metabolizing enzymes may vary among individuals, this does not indicate that critical events in disease processes occurring after exposure to hazardous agents differ qualitatively in animals and humans. Similarly, the human carcinogen, diethylstilbestrol (DES), activates estrogen receptor in animals and in humans.

Shanks and Pyles, while critical of the use of experimentation on animals to predict human health hazards, offer no alternative approach to indentifying environmental or occupational carcinogens and to formulating strategies to eliminate or reduce human exposures. Waiting for high incidences of human cancers, which may take 30 or more years from the time of first exposure to clinical manifestation of disease, is not an acceptable method for identifying human carcinogens. For several agents that were shown to be carcinogenic in animals, human carcinogenicity was later confirmed when reliable epidemiological data became available, e.g., 1,3-butadiene, cadmium, diethylstilbestrol, formaldehyde, ethylene oxide, and vinyl chloride [7]. We believe it to be irresponsible to ignore health effects data derived from animal studies. Public health protective strategies are needed to avoid repeating mistakes of the past similar to that of the DES tragedy in which adverse health effects in animals were ignored and human use of this drug not banned until 1971, after the discovery of high rates of rare, clear-cell adenocarcinomas of the vagina and cervix in DES-exposed daughters [10].

Although precise quantification of the effectiveness of cancer prevention programs is extremely difficult, public health is best served when primary prevention actions are taken based on adverse health effects identified in studies in animals or in humans. Trans species extrapolations of health risks could be improved with increased information on the range and distribution of factors affecting responses in human populations. While no animal model will allow a precise estimation of cancer risk in all humans, Shanks and Pyles contend that laboratory animals are not useful models for evaluating potential human carcinogenicity. This view is contrary to that of all major public health agencies, including the International Agency for Research on Cancer [11], the US National Toxicology Program [12], and the US Environmental Agency [13], which have adopted the perspective that even in the absence of carcinogenicity data in humans, it is biologically plausible that agents for which there is sufficient evidence of carcinogenicity in experimental animals pose a carcinogenic risk to humans. This view is based largely on the fact that all known human carcinogens that have been studied adequately in experimental animals produce positive carcinogenic results [12]. For chemicals that are considered to be possible or probable human carcinogens based on animal data and/or mechanistic data, insufficient data are available from exposed human populations to make definitive determinations of causal relationships.

In spite of some disagreements, darwinian medicine can certainly contribute to a greater understanding of the role of genetic variability or gene expression differences in dose-response relationships across species and among susceptible subpopulations.

## Competing interests

The author(s) declare that they have no competing interests.

## Authors' contributions

The authors contributed equally to the writing of the manuscript.
